# A mixed-methods study of hospital-acquired infections in Mongolia

**DOI:** 10.1186/1753-6561-5-S6-O15

**Published:** 2011-06-29

**Authors:** B-E Ider, A Clements, J Adams, M Whitby, A Morton, T Muugolog

**Affiliations:** 1 School of Population Health , University of Queensland, Australia; 2 Princess Alexandra Hospital, Brisbane , Australia; 3 National Center for Communicable Diseases, Ulaanbaatar, Mongolia

## Introduction / objectives

Official reports of Mongolia indicate that hospital-acquired infections (HAI) occur in 0.01–0.05% of all hospital admissions. This is considerably lower than internationally reported rates. There have been no published HAI prevalence studies from Mongolia. We aimed to determine the first accurate estimate of HAI prevalence in two tertiary hospitals of Ulaanbaatar and to seek explanation for underreporting of HAIs in Mongolian hospitals.

## Methods

In 2008, a one-day survey examined all 933 inpatients in two hospitals. Cases of HAI were diagnosed using CDC (USA) definitions. Subsequently, 87 health professionals were recruited for 55 interviews and 4 group discussions. Perceived reasons and mechanisms of underreporting were identified.

## Results

Prevalence of HAI was 50/933 (5.4%) overall HAI, 0.3% for bloodstream infection, 1.3% for respiratory tract infection, 1.3%, for urinary tract infection and 1.4%, for other HAI. Among surgical patients, prevalence of surgical site infection was 3.9%. Participants in the qualitative study explained that underreporting of HAI is mainly a response to punitive performance evaluation by the Ministry of Health (MoH) and penalisation of hospitals and staff by the State Inspection Agency when HAI were detected.

## Conclusion

The prevalence of HAIs in two Mongolian tertiary hospitals is comparable with reports from some other developing countries. The MoH statistics underestimate the true burden of HAI in Mongolia. Inclusion of the overall HAI rate in the targeted performance indicator set and the use of strict control and penalisation of hospitals with reported HAI cases are factors that have contributed to gaming, which has resulted in deliberate, extreme under-reporting of HAI cases in Mongolian hospitals.

## Disclosure of interest

None declared.

